# In-Silico Analysis and Antidiabetic Effect of α-Amylase and α-Glucosidase Inhibitory Peptides from Lupin Protein Hydrolysate: Enzyme-Peptide Interaction Study Using Molecular Docking Approach

**DOI:** 10.3390/foods11213375

**Published:** 2022-10-26

**Authors:** Gbemisola J. Fadimu, Asgar Farahnaky, Harsharn Gill, Olusegun A. Olalere, Chee-Yuen Gan, Tuyen Truong

**Affiliations:** 1 School of Science, RMIT University, GPO Box 2476, Melbourne, VIC 3001, Australia; 2 Analytical Biochemistry Research Center (ABrC), Universiti Sains Malaysia, University Innovation Incubator Building, SAINS@USM Campus, Bayan Baru 11900, Penang, Malaysia; 3 School of Science, Engineering and Technology, RMIT Vietnam, Ho Chi Minh City 700000, Vietnam

**Keywords:** lupin protein hydrolysate, bioactive peptide, α-amylase, α-glucosidase, LC-MS QTOF

## Abstract

The use of natural ingredients for managing diabetes is becoming more popular in recent times due to the several adverse effects associated with synthetic antidiabetic medications. In this study, we investigated the in vitro antidiabetic potential (through inhibition of α-glucosidase (AG) and α-amylase (AA)) of hydrolysates from lupin proteins pretreated with ultrasound and hydrolyzed using alcalase (ACT) and flavourzyme (FCT). We further fractionated ACT and FCT into three molecular weight fractions. Unfractionated ACT and FCT showed significantly (*p* < 0.05) higher AG (IC_50_ value = 1.65 mg/mL and 1.91 mg/mL) and AA (IC_50_ value = 1.66 mg/mL and 1.98 mg/mL) inhibitory activities than their ultrafiltrated fractions, where lower IC_50_ values indicate higher inhibitory activities. Then, ACT and FCT were subjected to peptide sequencing using LC-MS-QTOF to identify the potential AG and AA inhibitors. Molecular docking was performed on peptides with the highest number of hotspots and PeptideRanker score to study their interactions with AG and AA enzymes. Among the peptides identified, SPRRF, FE, and RR were predicted to be the most active peptides against AG, while AA inhibitors were predicted to be RPR, PPGIP, and LRP. Overall, hydrolysates prepared from lupin proteins using alcalase and flavourzyme may be useful in formulating functional food for managing diabetics.

## 1. Introduction

Diabetes, a chronic metabolic disease, is one of the leading causes of premature mortality in the world today. According to the International Diabetes Federation (IDF), one in ten adults has diabetes globally, implying that over 537 million people live with this chronic disease. This number is projected to increase dramatically to over 643 million by 2030 and 783 million by 2045 [1]. In 2021 alone, 6.7 million people died worldwide due to diabetes-related problems. Diabetes is a chronic condition that occurs when the organ responsible for insulin production (pancreas) stops making insulin or is unable to utilize the little it produces. Various types of diabetes, including gestational, type-1-diabetes, and type-2-diabetes, have been identified. However, approximately 90% of all diagnosed and reported cases of diabetes are type-2-diabetes and occur when insulin production is inadequate or when there is peripheral resistance to the action of insulin. One of the effective strategies for managing this condition involves using α-amylase and α-glucosidase inhibitors as antidiabetic medications [2]. This approach is effective since α-amylase and α-glucosidase enzymes are responsible for the degradation of carbohydrates into glucose units, causing blood sugar to rise. Regulation of postprandial hyperglycemia may therefore be achieved by inhibiting these enzymes and ultimately delaying glucose absorption.

Several antidiabetic agents, including miglitol, emiglitate, acarbose, and voglibose, have been developed over the years, are commercially available, and are effective as antidiabetic drugs [3]. However, continuous administration of these medications has been reported to have side effects, such as headache, nausea, and dizziness [4], that may aggravate other medical conditions in the body including cardiovascular events [5]; hence, there is a need to search for safer alternatives with no adverse effects and good drug profiles [4]. Bioactive peptides (BPs) derived from enzymatically hydrolysed food proteins, which are natural ingredients, have been found to be a safer alternative to synthetic antidiabetic drugs [6,7]. BPs can be derived from different food protein sources. However, animal protein, particularly milk, is the most studied food protein for the generation of BPs. Recently, the use of plant proteins in BP production has been gaining much research attention owing to their availability, cost-effectiveness, and high demand for BPs from vegetable sources [4]. Soybean is the most studied plant protein for BP production due mostly to its popularity and protein profile. However, using other plant proteins with comparable protein profiles is becoming more popular.

Lupin (*Lupinus angustifolius* L.) is a highly sustainable leguminous bean with a protein profile comparable to soybean. It has a very high protein content (33.9–43.4%) and less competition for direct consumption like soybean. Enzymatic hydrolysis of lupin proteins has been reported to yield hydrolysates with antihypertensive, antidiabetic, antioxidant, and antimicrobial activities [8,9]. In addition, the application of novel technologies, including the application of ultrasound to the proteins prior to enzymolysis, have been reported to yield hydrolysates with improved biological activities, owing largely to alterations of protein structure and causing the release of more peptides with better bioactivity. Our recent studies [4,10] about the biological activity of lupin proteins revealed that ultrasonication of lupin proteins prior to hydrolysis with alcalase, flavourzyme, and protamex yielded hydrolysates with strong antioxidant, antihypertensive, and antidiabetic activities.

However, information about the amino acid sequence of BPs with potential α-amylase and α-glucosidase inhibitory activities from enzymatic hydrolysis of lupin proteins using an in-silico approach is scarce and needs to be explored. Furthermore, information about the role of ultrasound pretreatment in the release of novel α-amylase and α-glucosidase inhibitory peptides from enzymic hydrolysed lupin proteins is not available in the literature. Previous studies on BP generation from lupin proteins only focused on hydrolysis of the protein using animal and vegetable enzymes without any pretreatment [11,12,13]. To the best of our knowledge, studies about the impact of ultrasound pretreatment prior to enzymatic hydrolysis of lupin proteins with alcalase and flavourzyme on peptides derived from lupin proteins and their inhibitory potential against the enzymes associated with diabetes (α-amylase and α-glucosidase) is not available in published form. Application of ultrasound pretreatment to lupin proteins followed by hydrolysis using alcalase and flavourzyme could generate novel peptides with enhanced inhibitory activity against the enzymes used for managing diabetes. Therefore, the aim of this study was to characterize the α-amylase and α-glucosidase inhibitory properties of the lupin protein hydrolysate, prepared using ultrasonicated lupin proteins and hydrolysed with alcalase and flavourzyme. Furthermore, the potent antidiabetic peptides were identified using LC-MS-QTOF. Additionally, molecular mechanisms for interaction between the potent antidiabetic peptides and the enzymes were analysed via in silico structural activity relationship using the molecular docking approach.

## 2. Materials and Methods

### 2.1. Materials and Chemicals

Flavourzyme (P6110: 500 U/g; EC 232-752-2 from *Aspergillus oryzae*), alcalase (P4860: ≥2.4 U/g; EC 3.4.21.62 from *Bacillus licheniformis*, Subtilisin A), α-amylase (≥1000 units/mg; EC 232-565-6 from the porcine pancreas), α-glucosidase (≥10 units/mg; EC 232-604-7 from *Saccharomyces cerevisiae*), 4-Nitrophenyl-β-d-glucopyranoside (ρNPG), 3,5-dintrosalicylic acid (DNSA), and starch (EC 232-679-6) were procured from Sigma Aldrich (Castle Hill, NSW, Australia). Membrane sheets used for ultrafiltration (Synder flat sheet membrane MT, ST and XT) were procured from Sterlitech Corporation (Auburn, WA, USA). Precast gel, precision plus standard, Coomassie blue, 2-mercaptoethanol, Tris/Glycine/SDS buffer, and Laemmli sample buffer were purchased from Bio-Rad Laboratories (Gladsville, NSW, Australia). Lupin protein isolate (LPI) was purchased from Prolupin GmbH (Grimmen, Germany) and kept at 4 °C prior to analysis.

### 2.2. Preparation of Lupin Protein Hydrolysates (LPH)

Preparation of LPH was performed using the method of Fadimu et al. [14]. A 10% (*w*/*v*) LPI solution was made in a beaker using MilliQ water, with continuous stirring for approximately 30 min at ambient temperature. Prior to enzymatic hydrolysis, the solution was ultrasonicated using Bandelin Sonoplus Ultrasonic Homogenizer HD3400 (Berlin, Germany) with 400 W fixed power, 20 kHz frequency, and a 25 mm diameter probe at 60% amplitude. The sonication process was performed for 5 and 10 min at an ultrasonic intensity of 52.24 W/cm^2^ and 104.5 W/cm^2^, respectively. To maintain an ambient temperature, the beaker was placed in an icebox. The solution was then adjusted to the optimum temperature (60 °C for alcalase and 50 °C for flavourzyme) and pH (8.0 for alcalase and 6.0 for flavourzyme) of the proteolytic enzymes and inoculated with the enzymes using a 3% enzyme/substrate ratio. Hydrolysis was allowed to proceed for 4 h and was terminated after 4 h by placing the solution in hot water at 95 °C for 15 min. The hydrolysate was then centrifuged, freeze-dried, and stored at −20 °C for further studies. This process yielded two hydrolysate samples: alcalase hydrolysate (ACT) and flavourzyme hydrolysate (FCT). Three batches of the hydrolysates were prepared, and experiments were performed in triplicates.

### 2.3. Fractionation of the Hydrolysate Samples

Protein hydrolysate prepared from the steps above (ACT and FCT) were fractionated into 3 molecular weight fractions (molecular weight cut-off (MWCO) of 1, 5, and 10 kDa) using the SEPA CF Cell membrane Filtration System (Sterlitech Corporation, Auburn, WA, USA). During the ultrafiltration process, the pressure of 25 bar and 3.5 LPM was maintained at a temperature of 15 °C. Six ultrafiltrated fractions were obtained for ACT (A1 kDa, A5 kDa, and A10 kDa) and FCT (F1kDa, F5 kDa, and F10 kDa) corresponding to the MWCO used. All fractionated samples were freeze-dried and kept at −20 °C for analysis. 

### 2.4. Sodium Dodecyl Sulphate-Polyacrylamide Gel Electrophoresis (SDS-PAGE) Analysis

SDS-PAGE analysis was carried out using precast polyacrylamide gel (4–20%) using the method of Laemmli [15] as described [10]. The hydrolysate samples and their fractions were dissolved in Milli-Q water. The sample buffer was prepared by mixing 2-mercaptoethanol (50 µL) and Laemmli sample buffer (950 µL) solutions. Then, the sample buffer and protein samples were mixed in a ratio of 1:1, heated for 5 min at 95 °C, loaded into precast gels, and run on a Mini-protean II system (Bio-Rad Laboratories, Hercules, CA, USA) for 45 min at a constant voltage of 120 V. Then, the gel was removed, stained with Coomassie brilliant blue for 24 h, and then destained using water/methanol/acetic acid solution. Molecular weight estimation was performed using a molecular weight marker of 10 to 250 kDa.

### 2.5. Determination of α-Amylase Inhibitory Activity

The method of Wickramaratne et al. [16], as previously described by Fadimu, Gill, Farahnaky, and Truong [4], was employed to determine the α-amylase inhibitory activity of the LPHs and their fractions. The protein samples (1–5 mg/mL) and α-amylase enzyme (2 units/mL) were dissolved in phosphate buffer (0.02 M). About 200 µL of the protein samples and their fractions were mixed with 200 µL α-amylase enzyme solution. The mixture was incubated for 10 min at 30 °C. This was followed by the addition of 200 µL starch solution (1% *w*/*v*) and equilibrated for 3 min. Afterward, 200 µL of Dinitrosalicylic acid (DNSA) reagent was added to the mixture and then boiled for 5 min. Finally, the reaction mixture was cooled to room temperature, followed by dilution with Milli-Q water (5 mL). The absorbance of the final reaction mixture was measured using a plate reader (FLUOstar Omega BMG Labtech GmbH, Ortenberg, Germany) at 540 nm. α-amylase inhibitory activity was calculated using the equation below:

$$\alpha -\mathrm{amylase}\;\mathrm{inhibition}\;\left(\%\right)=\frac{\left(\mathrm{Absorbance}\;\mathrm{of}\;\mathrm{control}\right)-\left(\mathrm{Absorbance}\;\mathrm{of}\;\mathrm{sample}\right)}{\mathrm{Absorbance}\;\mathrm{of}\;\mathrm{control}}\times 100$$

IC_50_ (concentration of peptides that inhibited 50% of α-amylase enzyme) value was estimated by plotting α-amylase inhibition (%) against sample concentration (mg/mL).

### 2.6. Determination of α-Glucosidase Inhibitory Activity

Alpha-Glucosidase inhibitory activity was measured using the method of Lankatillake et al. [17] as described by Fadimu, Gill, Farahnaky, and Truong [4]. The samples (50 µL) and α-glucosidase enzyme (30 µL) were added to a 96-well plate. The mixture was kept in the dark and incubated at 37 °C for 10 min. Following incubation, the reaction process was initiated by adding 20 µL of 4-nitrophenyl-β-d-glucopyranoside (5 mM) solution to the wells containing the enzyme and samples. The mixture was then incubated at 37 °C for 20 min. Afterward, the absorbance of the mixture was measured at 405 nm using a plate reader (FLUOstar Omega BMG Labtech GmbH, Ortenberg, Germany). The inhibitory activity was estimated using the equation below:

$$\alpha -\mathrm{glucosidase}\;\mathrm{inhibition}\;\left(\%\right)=\frac{\left(\mathrm{Absorbance}\;\mathrm{of}\;\mathrm{control}\right)-\left(\mathrm{Absorbance}\;\mathrm{of}\;\mathrm{sample}\right)}{\mathrm{Absorbance}\;\mathrm{of}\;\mathrm{control}}\times 100$$

IC_50_ (concentration of peptides that inhibited 50% of α-glucosidase enzyme) value was estimated by plotting α-glucosidase inhibition (%) against sample concentration (mg/mL).

### 2.7. Identification of Peptides Implied in α-Amylase and α-Glucosidase Inhibitory Activities Using Liquid Chromatography-Mass Spectrometry of Quadrupole Time-of-Flight (LC-MS QTOF) and In-Silico Approach

LC-MS-QTOF was employed for the possible identification of peptides in samples with the highest inhibitory activities (ACT and FCT) using the method of Sarah et al. [18]. Firstly, the hydrolysate samples (1 mg) were solubilized in 1 mL of deionized water with formic acid (0.1%). Two solvents, A (demineralized water containing 0.1% formic acid) and B (LCMS grade acetonitrile containing 0.1% formic acid), were used as the mobile phases. Separation of peptides was performed using Advance Bio Peptide Map (C18 column; 2.1 × 100 mm, 2.7 µm particles, Agilent (Santa Clara, CA, USA)) at a 15 µL/min flow rate. Analysis of peptides were performed using electrospray ionization-quadrupole time-of-flight system (ESI-QTOF, Agilent 6520) using the following settings: (a) ion source: 3.5 kV, (b) collision energy: 6 V/100 Da (offset −2), and (c) mass range: 100–2000 *m*/*z*. The mass spectrometric data obtained were then analysed using Peaks studio version 6.0 having average local confidence (ALC) exceeding 80%. Analysis of the bioactivity of the identified peptides was performed using the Peptide Ranker server at http://distilldeep.ucd.ie/PeptideRanker/ (accessed on 15 April 2022). Peptides with Ranker scores above 0.5 were selected for further analysis. The molecular mechanism of α-amylase and α-glucosidase inhibition by the selected peptides was established using PepSite 2, a web-based (http://pepsite2.russelllab.org/ (accessed on 15 April 2022)) platform for checking in silico molecular interaction between enzymes and peptides. Then, the lysosomal α-glucosidase (PDB ID: 5NN3) and α-amylase (PDB ID: 1SMD) crystal structures were downloaded from the PepSite 2 web-based program. A significant level below 5% (*p* < 0.05) was used to select the most potent peptides and their potential binding sites.

### 2.8. Molecular Docking

The 3D structures of lysosomal α-glucosidase (PDB ID: 5NN3) and α-amylase (PDB ID: 1SMD) were downloaded from the Ressource Parisienne en Bioinformatique Structurale (RPBS) website (http://www.rcsb.org (accessed on 16 April 2022)). De novo peptide structure was obtained from the Pep-Fold 3 server (https://bioserv.rpbs.univ-paris-diderot.fr/services/PEP-FOLD3/#overview (accessed on 16 April 2022)) [19]. Pre-processed peptide structures were later optimized and minimized to generate geometrically stable structures [20]. Docking of ACE peptides into the protein structure was performed using the Haddock server (http://wenmr.science.un.nl/haddock2.4/ (accessed on 17 April 2022)) as described by Honorato et al. [21]. The interaction of α-glucosidase and α-amylase enzymes with the selected peptides (PPGIP, RPR, LRP, SPRRF, FE, and RR) was examined using docking experiments.

### 2.9. Statistical Analysis

All measurements were conducted in triplicate, and data were reported as mean ± standard deviation. Analysis of variance (ANOVA) was performed using SPSS statistical software (26.0 version, Michigan State University, East Lansing, MI, USA). Mean separation was done using Duncan’s Multiple Range Test (DMRT), and significant differences were defined as *p* < 0.05.

## 3. Results and Discussions

### 3.1. SDS-PAGE Analysis

**Figure 1:**
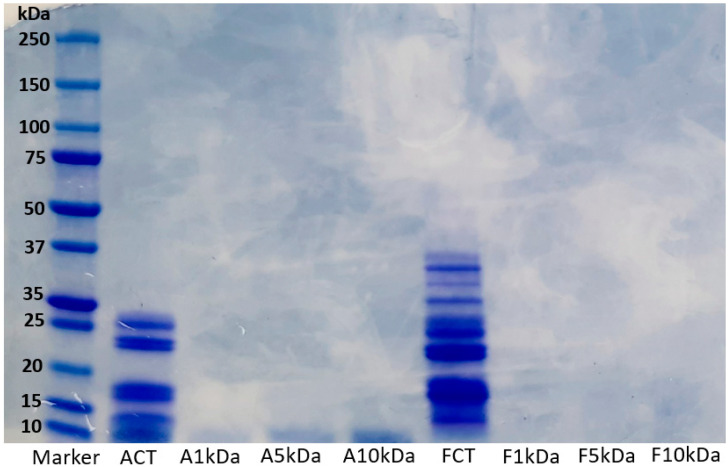
Electrophoretic pattern of LPHs generated using alcalase and flavourzyme and their ultrafiltrated fractions. ACT, unfractionated alcalase hydrolysate; FCT, unfractionated flavourzyme hydrolysate; A1 kDa and F1 kDa, <1 kDa fraction; A5 kDa and F5 kDa, <5 kDa fraction; A10 kDa and F10 kDa, <10 kDa fraction.

Short-chain proteins or peptides resulting from proteolysis reactions have different molecular weights. The degradation pattern of lupin protein hydrolysates generated using alcalase (ACT) and flavourzyme (FCT) and their various ultrafiltrated fractions (A1 kDa, A5 kDa, A10 kDa, F1 kDa, F5 kDa, and F10 kDa) are presented in Figure 1. Furthermore, the degree of hydrolysis and protein content of ACT and FCT hydrolysates are presented in Supplementary Table S1. Overall, the enzymatic hydrolysis reduced the molecular weight of the proteins. Generally, the hydrolysate prepared using alcalase contained lower molecular weight proteins compared with the flavourzyme hydrolysate. In the original flavourzyme hydrolysate, the thick bands observed from the middle towards the bottom of the gel is an indication that the peptides generated were retained towards the low molecular weight range, whereas more extensive hydrolysis has occurred in the alcalase hydrolysate. The slight variations observed may be linked to differences in specificity and cleavage pattern of alcalase and flavourzyme. This result is consistent with our previous study, where variable degradation of lupin proteins was observed after enzymatic hydrolysis with alcalase and flavourzyme [4]. At molecular weights above 10 kDa, no visible band was observed in the ultrafiltrated fractions. This implies that the ultrafiltration process separated the proteins into the desired molecular weight portions of 1 kDa, 5 kDa, and 10 kDa. Overall, it can be deduced that the molecular weight of peptides generated from lupin proteins depends on the type of enzyme used.

### 3.2. Inhibition of the α-Amylase Inhibitory Activity

Diabetes is a chronic condition characterized by high glucose levels in the blood due to the inability of the pancreas to produce enough insulin needed to facilitate the transportation of glucose into the cells. Glucose is the final product of carbohydrate metabolism in the body. Delaying carbohydrate hydrolysis into glucose by inhibiting the critical enzyme required for the reaction has been an effective approach for managing blood glucose levels. Alpha-amylase is one of the enzymes that hydrolyze starch into glucose, and blood glucose levels may be effectively managed through its inhibition. The result of IC_50_ values of lupin protein hydrolysates and their ultrafiltrated fractions (Table 1) showed that the ultrafiltration of LPH caused a reduction in the α-amylase inhibitory property of its hydrolysates. The highest α-amylase inhibitory activity was observed in unfractionated hydrolysates generated using alcalase (ACT) (IC_50_ value = 1.66 mg/mL), followed by unfractionated hydrolysates from flavourzyme (FCT) (IC_50_ value = 1.98 mg/mL). This result is in accordance with previous studies which reported that hydrolysates generated using alcalase had significantly higher α-amylase inhibitory (lower IC_50_ value) ability than those prepared using flavourzyme, based on IC_50_ values [4,10].

In this study, ultrafiltration of ACT into 1 kDa, 5 kDa, and 10 kDa molecular weight fractions increased the IC_50_ value from 1.66 mg/mL to 4.87, 3.58, and 3.85 mg/mL, respectively. A similar trend was observed in the α-amylase inhibitory property of hydrolysates generated using flavourzyme. A significant increase in the IC_50_ value of FCT (1.98 mg/mL) to 3.52, 4.38, and 3.19 mg/mL was observed after ultrafiltration into 1 kDa, 5 kDa, and 10 kDa molecular weight fractions, respectively. The variations observed in the potency of the samples against α-amylase could be associated with the differences in their amino acid and peptide composition, as reported in the previous study [4]. As shown in Figure 1, the unfractionated hydrolysates ACT and FCT contained all the peptides generated during the proteolysis. The ultrafiltration process separated the peptides into 1 kDa, 5 kDa, and 10 kDa fractions and, in each case, retained only peptides below the selected molecular weight cutoff and caused a decrease in α-amylase inhibitory activity. This suggests that α-amylase inhibition is due to the synergistic effects of all peptides present in the sample. This result corroborates with those of Awosika and Aluko [22], who reported that unfractionated pea protein hydrolysates exhibited higher α-amylase inhibitory activity than their fractionated fractions. On the contrary, Famuwagun, Alashi, Gbadamosi, Taiwo, Oyedele, Adebooye, and Aluko [2] and Kamran, Phillips, and Reddy [9] reported a significant increase in IC_50_ values upon fractionation. Fractionation of protein hydrolysates into various molecular weight fractions is not a guarantee for improved activity [23], but in most cases it is important to determine the peptide fraction that contributes to the observed activity. 

### 3.3. Inhibition of the α-Glucosidase Inhibitory Activity

Inhibition of α-glucosidase is one of several approaches for managing blood glucose levels in diabetic patients. This inhibition often disrupts the catalytic activity of the enzyme, and the overall effect is the reduction in postprandial blood glucose level due to a delay in glucose absorption. The antidiabetic effects of lupin protein hydrolysates have been studied but the peptides responsible and the mechanisms behind the impact remain unknown [4]. The IC_50_ values for α-glucosidase inhibitory activity of hydrolysates and their ultrafiltrated fractions prepared from lupin proteins using alcalase and flavourzyme are presented in Table 1. Alcalase hydrolysate (ACT) (IC_50_ value of 1.65 mg/mL) had higher α-glucosidase inhibitory activity than flavourzyme hydrolysate (FCT) (IC_50_ value of 1.91 mg/mL). Upon fractionation into 1, 5, and 10 kDa molecular weight fractions, a general reduction in α-glucosidase inhibitory activity was observed. Among the fractions, the highest α-glucosidase inhibitory activity was recorded for the 10 kDa fraction of alcalase hydrolysate (IC_50_ value of 3.78 mg/mL), closely followed by the 10 kDa fraction of flavourzyme hydrolysate (IC_50_ value of 4.07 mg/mL). The lowest inhibitory activity was recorded in the 1 kDa fraction of ACT and FCT (IC_50_ values of 4.51 and 4.37 mg/mL, respectively) and 5 kDa fraction of FCT (IC_50_ values of 4.49 mg/mL). The low inhibitory activity observed in the ultrafiltrated fractions could be linked to a reduction in the composition of peptides in the various fractions since FCT and ACT contained all the peptides generated during the hydrolysis. In contrast, their ultrafiltrated counterparts possessed only a fraction of the peptides. These results indicate that the fractionation process decreased the concentration of peptides, thereby causing a reduction in their IC50 values. A similar observation has been reported for ultrafiltrated fractions of protein hydrolysates [24,25]. Overall, the results presented in this study indicate that hydrolysates with antidiabetic effects could be generated from ultrasonicated lupin proteins using alcalase and flavourzyme. Those effects are due to the synergistic action of all peptides in the hydrolysate.

### 3.4. Identification and Selection of α-Amylase and α-Glucosidase Inhibitory Peptides from Selected Lupin Protein Hydrolysates

Unfractionated lupin protein hydrolysates generated using alcalase (ACT) and flavourzyme (FCT) were selected for further studies due to their relatively higher IC_50_ values compared with their ultrafiltrated fractions (Table 1). Peptide identification was performed using LC-MS-QTOF. A total of 38 and 67 peptides were identified in ACT and FCT, respectively (Supplementary Tables S2 and S3). In both samples, peptides with a Peptide ranker score above 0.5 were selected and subjected to in silico structure relationship analysis with α-amylase and α-glucosidase enzymes using the Peptide ranker server (http://distilldeep.ucd.ie/PeptideRanker/ (accessed on 15 April 2022)) (Table 2, Table 3, Table 4 and Table 5). In addition to the Peptide Ranker score, identified peptides were screened and ranked according to their Pepsite2 *p*-value, reactive residues, and the number of potential binding sites of the peptides to ensure that they meet the criteria for being biologically active. Pepsite2 is a web-based server commonly employed for predicting interactions between proteins and peptides [26]. Peptides with very low Pepsite2 *p*-values (*p* < 0.05) are more likely to bind with the active site of α-amylase and α-glucosidase, causing them to lose their activity through the blockage of the substrate and catalytic sites. In total, 16 peptides were found to be active inhibitors of α-glucosidase (Table 2 and Table 3) and 14 for α-amylase (Table 4 and Table 5) based on Peptide Ranker score, Pepsite2 *p*-values, and several potential binding sites. Toxicity predictions according to ToxidPred also indicate that all the selected peptides were non-toxic.

### 3.5. Molecular Interaction of Lupin Protein-Derived α-Glucosidase Inhibitory Peptides with α-Glucosidase

According to Table 2, 16 peptide sequences from alcalase hydrolysate were predicted to have the ability to disrupt the activity of α-glucosidase (*p* < 0.05). According to Hermans et al. [27], the three most important amino acid residues in the catalytic site of α-glucosidase responsible for its activity are Trp516, Asp518, and Asp513. We predicted the binding site residues of the identified α-glucosidase inhibitors using molecular docking as described by Bruckmann et al. [28]. Interestingly, Trp376, Trp516, Asp616, Met519, Asp404, Ile441, Asp518, Asp616, His674, Arg600, and Phe649 were found to be the main residues in α-glucosidase that interact with the identified peptide. Kamal et al. [29] and Roig-Zamboni et al. [30] reported similar results when they studied the molecular interaction between α-glucosidase and α-glucosidase inhibitory peptides. Accordingly, peptides SPRRF, FE, and RR from alcalase hydrolysates were predicted to have the highest potency against α-glucosidase in comparison to other peptides due to their abilities to bind 11 residues of the α-glucosidase enzyme (Table 2). In addition, basic and hydrophobic amino acids, such as arginine, proline, and phenylalanine, which have been indicated to be an essential contributor to the α-glucosidase inhibitory potential of peptides, could have enhanced their potency against α-glucosidase. Other peptides that could bind up to 10 binding sites were AIPINNPGKL, AIPPGIPY, and LRL. Peptides with the lowest binding sites were FP and RW, binding only to 3 and 6 sites, respectively, on α-glucosidase.

Similarly, 14 peptide sequences from the flavourzyme hydrolysate (FCT) were predicted to have the capability to hinder the catalytic activity of α-glucosidase (Table 3). In this case, peptides PPGIP, RPR, and LRP were predicted to be the most active inhibitors of α-glucosidase owing to their abilities to bind 11 residues of the α-glucosidase enzyme. The peptide PPGIP could bind Trp376, Asp404, Ile441, Asp518, Met519, Arg600, Asp616, and Phe649 (Table 3), while RPR and LRP could bind Trp516 in addition to those bound by PPGIP. Taken together, the α-glucosidase inhibitory peptides identified in this study could inhibit the activity of the α-glucosidase enzyme due to their ability to bind over 10 residues on the enzyme, which suggests that they could hinder the hydrolysis of carbohydrates into glucose. This delay could potentially reduce the rate of absorption and transportation of glucose into the bloodstream. Hence, lupin hydrolysates that have been ultrasonicated and hydrolysed using alcalase and flavourzyme could be helpful as a functional ingredient in the formulation of nutraceuticals for managing diabetes.

### 3.6. Molecular Interaction of Lupin Protein-Derived α-Amylase Inhibitory Peptides with α-Amylase

The potential molecular interaction between the α-amylase enzyme and peptides from alcalase and flavourzyme hydrolysates are presented in Table 4 and Table 5. All the peptides exhibited strong binding potential against α-amylase and were predicted as potential α-amylase inhibitors. To establish how the identified peptides interact with α-amylase, we performed molecular interaction studies using the Pepsite2 computational approach to predict the way the peptides bind onto the active site of the α-amylase enzyme. Structurally, the α-amylase enzyme comprises three distinctive domains: A, B, and C, where interaction between the substrate and the enzyme occurs. Domain A accounts for the vast majority of the domains. The substrate-binding sites are present in this domain and are linked to domain B via its carboxyl end. The stability of the enzyme and substrate specificity is maintained by domain B [31], while domain C is responsible for maintaining the stability of the enzyme’s catalytic sites [32]. Studies performed on the α-amylase enzyme indicate that seven major amino acid residues are involved in its catalytic process. These are Trp59, Trp58, Tyr62, His299, Asp96, His305, Asp197, and Asp300. These amino acids are known to form the catalytic triad and are potential inhibitory sites of the α-amylase enzyme [33]. Therefore, we believe that the binding of peptides to these residues could regulate carbohydrate metabolism.

To predict the way peptides bind onto the active site of the α-amylase enzyme, we used the Pepsite2 computational approach. To achieve this, we calculated the *p*-value and number of hotspots using statistical significance (Table 4). In this study, we obtained a very low *p*-value for all the peptides, indicating that Pepsite2 accurately predicted the peptide-protein binding interaction. Among the peptides from the alcalase hydrolysate, SPRRF (Trp59, Asp96, Trp58, Tyr62, His299, and Asp300), MLLL (Trp58, Trp59, Tyr62, Asp96, Hiss299, and Asp300), and AIPPGIPY (Trp58, Trp59, Tyr62, His299, Asp300, His305) could bind 6 sites or residues on α-amylase. Other peptides such as RW, PMLL, ML, AIPINNPGKL, HSDADFIL, RLL, LR, LRL, and LLPH also showed high α-amylase inhibitory potential by binding 5 active site residues. However, peptide FP could only bind to 3 sites (Trp58, Trp59, and Asp300) of the catalytic triad. This agrees with the study by Siow and Gan [34], as they reported amino acids Trp58, Trp59, His299, Asp300, Asp197, His305, and Tyr62 as major amino acids in the catalytic triad of α-amylase.

Among peptides identified in the flavourzyme hydrolysate, ML, LP, and RPR demonstrated high α-amylase inhibitory capacity as they bind 5 hotspots on the triad (Table 5). This correlates with the lower α-amylase inhibitory activity value of this hydrolysate (IC50 value 1.98 mg/mL). Others could bind only 4 active sites of the triad. Altogether, the peptides SPRRF, MLLL, and AIPPGIPY from alcalase hydrolysates and ML, LP, and RPR from flavourzyme hydrolysates tend to be potential inhibitors that could block the active site of α-amylase and prevent postprandial hyperglycaemia by delaying hydrolysis of dietary carbohydrate into glucose.

### 3.7. Mechanism of Molecular Binding and Molecular Docking of Novel Peptides with α-Amylase and α-Glucosidase

**Figure 2:**
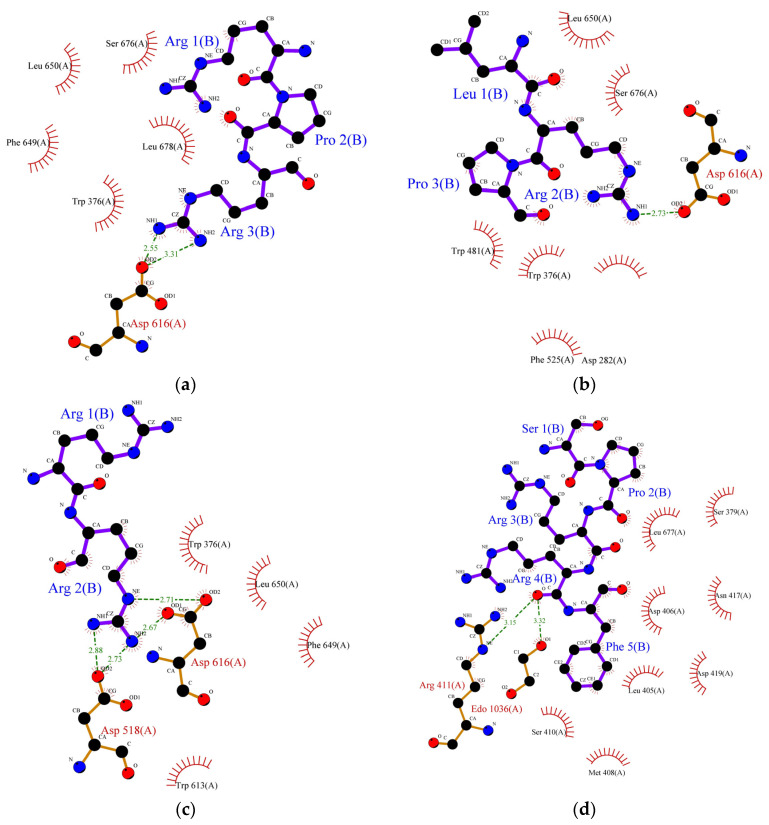

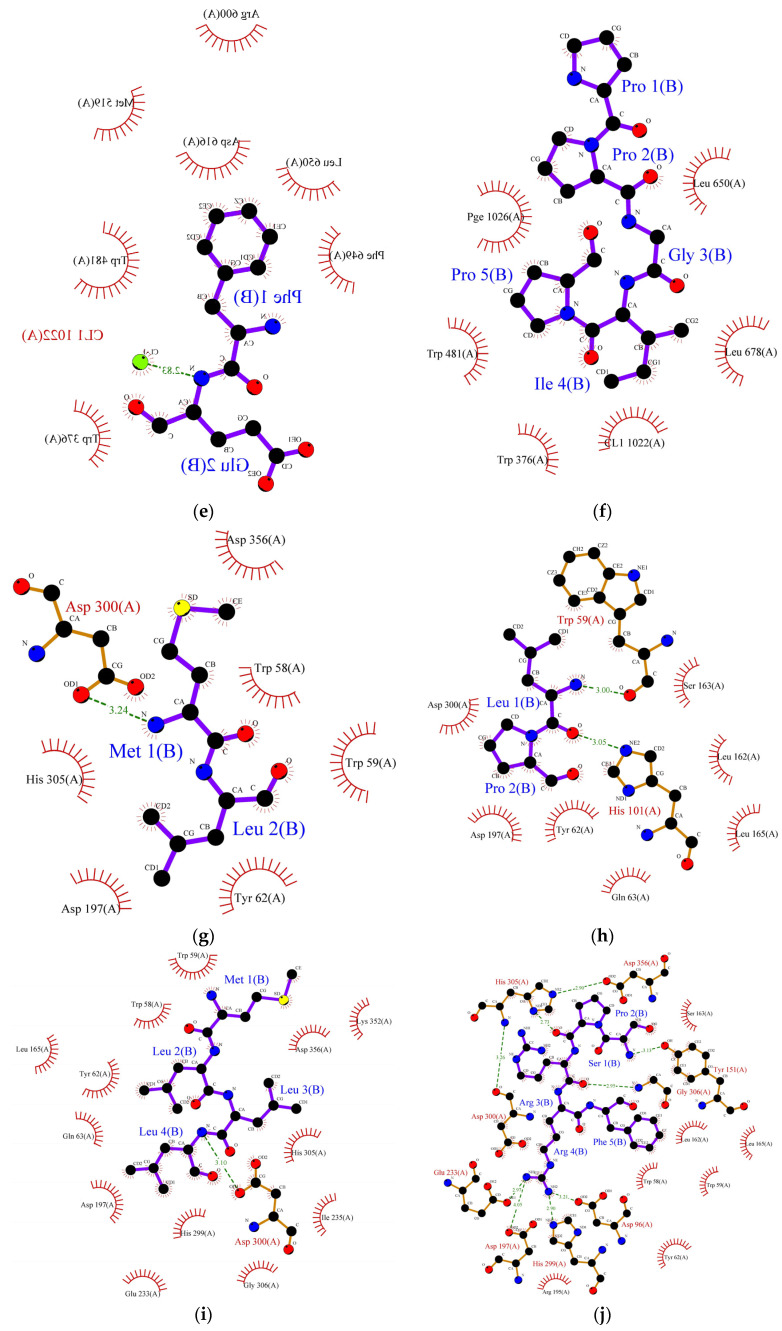

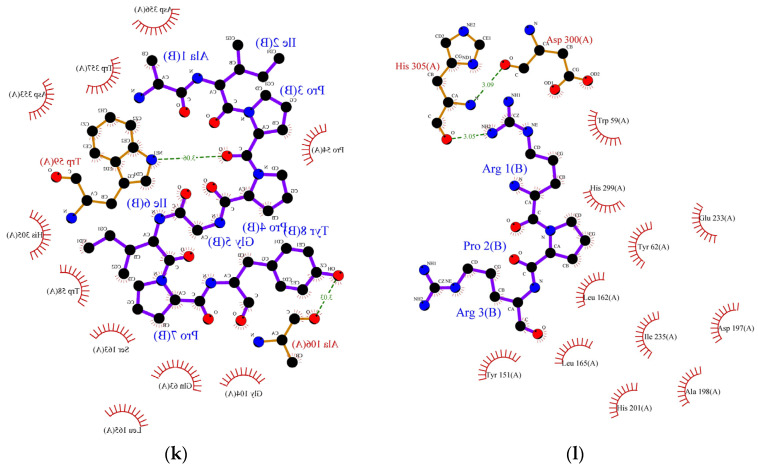
Protein ligand binding interaction between (**a**) RPR, (**b**) LRP, (**c**) RR, (**d**) SPRRF, (**e**) FE, and (**f**) PPGIP and α-glucosidase enzyme as well as (**g**) ML, (**h**) LP, (**i**) MLLL, (**j**) SPRRF, (**k**) AIPPGIPY, and (**l**) RPR and α-amylase enzyme. The blue ball, nitrogen atom; yellow ball, sulphur atom; red ball, oxygen atom; black ball, carbon atom; purple line, peptide; green dotted line with a number, hydrogen bond, and length of bond; red eyelashes, hydrophobic interaction.

Molecular docking is the most effective approach for identifying the binding patterns or forces behind protein-ligand complexes and is widely used in drug research [35]. Alpha-amylase and α-glucosidase inhibition are predominantly targeted for the management of type-II diabetes. These enzymes contain several catalytic active sites and the binding of inhibitors to these sites could prevent the formation of an enzyme-substrate complex. Alpha-glucosidase (EC 3.2.1.20) is one of the crucial enzymes involved in the hydrolysis of carbohydrates, such as starch, into glucose. This 20 kDa molecule catalyses the breakdown of the 1,4-glycosidic bond in starch and releases alpha glucose molecules in the process. Research involving inhibition of its catalytic activity is gaining lots of attention, particularly in pharmaceutical-related fields, since its inhibition could delay glucose uptake and therefore decrease postprandial blood glucose levels [36]. The exact mechanism of inhibition of α-glucosidase by peptides remains unknown, but it is generally believed that inhibition occurs when peptides bind to the enzyme’s active site and disrupt its catalytic activity. Earlier, we studied the correlation between the structure and activity of the identified peptides from lupin proteins. We observed that six peptides (SPRRF, FE, RR, PPGIP, LRP, and RPR) from alcalase and flavourzyme hydrolysates have a very strong potential to inhibit α-glucosidase by blocking the active site residues of the enzyme, thereby preventing hydrolysis of carbohydrate. The molecular-structural interaction between these peptides with α-glucosidase is presented in Figure 2a–f.

As indicated by molecular docking, all the peptides could easily bind on the active site of α-glucosidase with high binding energy (Table 6). The tripeptide RPR from the flavourzyme hydrolysate docked the active pocket of α-glucosidase with binding energy −6.1 kcal/mol, formed a hydrogen bond with the Asp616 residue, and interacted hydrophobically with the catalytic triad of α-glucosidase using Trp376 (Figure 2a). Using the same residue (Asp616) to form a hydrogen bond like RPR, LRP interacted with the catalytic residue of α-glucosidase using Trp376 with a slightly higher binding energy (−6.2 kcal/mol) (Figure 2b). The dipeptide RR formed interacted hydrophobically with Trp376 and Phe649 with binding energy −6.4 kcal/mol and formed hydrogen bonds using residues Asp616 and Asp518 (Figure 2c). Similarly, the pentapeptide SPRRF from ACT bound to the active site of α-glucosidase with a binding energy of −6.6 kcal/mol and formed hydrophobic interactions with catalytic residues Asp404. Among peptides identified in ACT, FE and PPGIP could not form hydrogen bonds. FE could form hydrophobic interactions using Phe649, Asp616, and Trp376, while PPGIP used only Trp376 for hydrophobic interactions with a catalytic triad of α-glucosidase.

In the case of α-amylase, Siow et al. [37] proposed that the mechanism of action for the inhibition of α-amylase by the peptides is based on the ability of the peptides to form a sliding barrier through the establishment of hydrogen bonds with the residues present around the substrate-binding region. All the identified peptides formed a hydrogen bond with the enzyme (Table 6). The dipeptide ML from FCT formed a hydrogen bond using Asp300 and interacted hydrophobically with the catalytic triad of α-amylase using Trp58, Trp59, and Tyr62 with a binding energy of −6.9 kcal/mol. The highest number of hydrogen-bond forming residues was recorded in pentapeptide SPRRF from the alcalase hydrolysate. This peptide formed a hydrogen bond using nine residues, His305, Asp356, Tyr151, Gly306, Asp96, His299, Glu233, Ap300, and Asp197 with −9.1 kcal/mol binding energy while interacting hydrophobically with catalytic pockets of α-amylase using Trp58, Trp59, and Tyr62. This interaction could have contributed to the higher IC_50_ value reported in the ACT (1.66 mg/mL) in comparison to FCT (1.98 mg/mL). Other α-amylase inhibitory peptides such as LP, MLLL, AIPPGIPY, and RPR also showed strong α-amylase inhibitory potential as they formed hydrogen bonds with at least one residue and interacted well with the substrate-binding pockets of α-amylase (Table 6).

## 4. Conclusions

Studies exploring the use of lupin protein hydrolysates as a functional ingredient for the development of nutraceuticals for managing lifestyle-related health conditions, such as diabetes, continue to gain considerable research attention. In this current study, we generated hydrolysates from lupin proteins using alcalase (ACT) and flavourzyme (FCT) and fractionated them into different molecular weight fractions. The IC50 values obtained showed that the original hydrolysates from alcalase and flavourzyme displayed higher inhibitory potentials towards α-glucosidase and α-amylase than their ultrafiltrated fractions. Lupin protein hydrolysates effectively inhibited the two enzymes involved in the management of diabetes (α-glucosidase and α-amylase) through disruption of enzyme-substrate interactions at the active site of the enzyme and delayed postprandial hyperglycaemia. Using an in-silico approach, peptides SPRRF, FE, RR from ACT and PPGIP, RPR, and LRP from FCT were predicted to be the most potent inhibitors of α-glucosidase. In contrast, MLLL, AIPPGIPY, and SPRRF from ACT and ML, LP, and RPR from FCT were predicted as the most active inhibitors of α-amylase, due to their abilities to bind several hotspots on carbohydrases. The mode of interaction between these peptides and the carbohydrases was explored using molecular docking, and the results indicated that the peptides have a very strong potential to bind to the catalytic site of the enzymes and inhibit them. Additionally, this study has established that peptides derived from lupin protein hydrolysates could bind to a higher number of bound residues on the active site of α-glucosidase in comparison to α-amylase. In summary, hydrolysates from lupin proteins generated using either alcalase or flavourzyme could be used as a functional ingredient in the development of nutraceuticals for diabetic patients. However, the potential activities of the identified peptides in their pure form should be validated in future studies by using in vivo, in vitro, and cell line assays.

## Figures and Tables

**Table 1:** IC_50_ values of α-amylase and α-glucosidase inhibitory activities of lupin protein hydrolysates generated using alcalase, flavourzyme, and their ultrafiltrated fractions.

Sample	IC_50_ Values (mg/mL)	
	α-Amylase	α-Glucosidase
ACT	1.66 ± 0.01 ^e^	1.65 ± 0.02 ^d^
A1kDa	4.87 ± 0.48 ^a^	4.51 ± 0.16 ^a^
A5kDa	3.58 ± 0.13 ^cd^	4.27 ± 0.15 ^ab^
A10kDa	3.85 ± 0.13 ^c^	3.78 ± 0.19 ^c^
FCT	1.98 ± 0.01 ^e^	1.91 ± 0.02 ^d^
F1kDa	3.52 ± 0.28 ^cd^	4.37 ± 0.02 ^a^
F5kDa	4.38 ± 0.01 ^b^	4.49 ± 0.02 ^a^
F10kDa	3.19 ± 0.04 ^d^	4.07 ± 0.04 ^b^

Values are mean ± standard deviation of triplicate determination. Values with different superscripts on the same column are significantly (*p* < 0.05) different. Abbreviations: ACT, unfractionated alcalase hydrolysate; FCT, unfractionated flavourzyme hydrolysate; A1kDa and F1kDa, <1 kDa fraction; A5 kDa and F5kDa, <5 kDa fraction; A10 kDa and F10 kDa, <10 kDa fraction.

**Table 2:** Biologically active peptides from a selected lupin protein hydrolysate (ACT) and their binding potential with α-glucosidase (5NN3) as a protein receptor.

Peptide Sequence	Peptide Ranker Score	PepSite2 *p*-Value	Reactive Residues in Peptide	Bound Residues of α-Glucosidase (5NN3)
**FP**	0.993916	0.0004915	F1, P2	Asp282, Trp376 *, Trp481, Met519 *, Phe525, Asp616 *
**RW**	0.978386	0.01098	R1, W2	Trp376 *, Asp404 *, Ile441 *, Trp481, Trp518, Asp518 *, Asp616 *, His674 *
**SPRRF**	0.919692	0.001218	S1, P2, R3, R4	Asp282, Trp376 *, Asp404 *, Ile441 *, Trp481, Trp516 *, Asp518 *, Met519 *, Phe525, Arg600 *, Asp616 *, Phe649 *, His674 *
**PMLL**	0.89733	0.004202	P1, M2, L3, L4	Asp282, Trp376 *, Asp404 *, Ile441 *, Trp481, Asp518 *, Met519 *, Phe525, Asp616 *
**ML**	0.894564	0.02483	M1, L2	Trp376 *, Asp404 *, Ile441 *, Trp481, Trp516 *, Asp518 *, Asp616 *, His674 *
**AIPINNPGKL**	0.807084	0.003297	P3, I4, N5, N6, P7, K9	Asp282, Trp376 *, Asp404 *, Ile441 *, Trp481, Trp516 *, Asp518 *, Met519 *, Phe525, Trp613 *, Asp616 *, Phe649 *, His674 *
**MLLL**	0.756994	0.04779	M1, L2, L3, L4	Asp282, Trp376 *, Asp404 *, Ile441 *, Trp481, Trp516 *, Asp518 *, Met519 *, Phe525, Asp616 *, Phe649 *, His674 *
**AIPPGIPY**	0.753808	0.008538	P3, P4, G5, I6, P7	Asp282, Asp404 *, Ile441 *, Trp481, Trp516 *, Asp518 *, Met519 *, Phe525, Arg600 *, Trp613 *, Asp616 *, Phe649 *, His674 *
**HSDADFIL**	0.683272	0.02868	H1, S2, D3, A4, D5, F6	Trp376 *, Asp404 *, Ile441 *, Trp481, Asp518 *, Met519 *, Phe525, Asp616 *, Phe649 *, His674 *
**RLL**	0.607206	0.02058	R1, L2, L3	Trp376 *, Asp404 *, Ile441 *, Trp481, Trp516 *, Asp518 *, Asp616 *, Phe649 *, His674 *
**FE**	0.589707	0.1065	F1, E2	Trp376 *, Asp404 *, Ile441 *, Trp481, Trp516 *, Asp518 *, Trp613 *, Asp616 *, Phe649 *, His674 *
**LR**	0.569984	0.009125	L1, R2	Trp376 *, Asp404 *, Ile441 *, Trp481, Trp516 *, Asp518 *, Asp616 *, Phe649 *, His674 *
**RR**	0.565498	0.001149	R1, R2	Trp376 *, Asp404 *, Ile441 *, Trp481, Trp516 *, Asp518 *, Met519 *, Arg600 *, Asp616 *, Phe649 *, His674 *
**LRL**	0.564172	0.03196	L1, R2, L3	Trp376 *, Asp404 *, Ile441 *, Trp481, Trp516 *, Asp518 *, Arg600 *, Trp613 *, Asp616 *, Phe649 *, His674 *
**SVPGCT**	0.501911	0.02842	S1, P3, G4, C5	Asp282, Trp376 *, Asp404 *, Ile441 *, Trp481, Asp518 *, Met519 *, Phe525, Arg600 *, Asp616 *, Phe649 *
**LLPH**	0.501446	0.001936	L1, L2, P3, H4	Asp282, Trp376 *, Asp404 *, Ile441 *, Trp481, Asp518 *, Met519 *, Phe525, Asp616 *, Phe649 *, His674 *

* Binding site for α-glucosidase inhibitors; peptides in bold are those selected for molecular docking.

**Table 3:** Biologically active peptides from a selected lupin protein hydrolysate (FCT) and their binding potential with α-glucosidase (5NN3) as a protein receptor.

Peptide Sequence	Peptide Ranker Score	PepSite2 *p*-Value	Reactive Residues in Peptide	Bound Residues of α-Glucosidase (5NN3)
**FP**	0.993916	0.0004915	F1, P2	Asp282, Trp376 *, Trp481, Met519 *, Phe525, Asp616 *
**ML**	0.894564	0.02483	M1, L2	Trp376 *, Asp404 *, Ile441 *, Trp481, Trp516 *, Asp518 *, Asp616 *, His674 *
**PPGIP**	0.838217	0.001023	P1, P2, G3, I4	Asp282, Trp376 *, Asp404 *, Ile441 *, Trp481, Asp518 *, Met519 *, Phe525, Arg600 *, Asp616 *, Phe649 *
**TF**	0.826678	0.01348	T1, F2	Trp376 *, Asp404 *, Ile441 *, Trp481, Asp518 *, Met519 *, Arg600 *, Asp616 *
**AIPINNPGKL**	0.807084	0.003297	P3, I4, N5, N6, P7, K9	Asp282, Trp376 *, Asp404 *, Ile441 *, Trp481, Trp516 *, Asp518 *, Met519 *, Phe525, Trp613 *, Asp616 *
**LP**	0.79612	0.001344	L1, P2	Asp282, Trp376 *, Trp481, Met519 *, Phe525, Asp616 *, Phe649 *
**RPR**	0.722632	0.0002172	R1, P2, R3	Asp282, Trp376 *, Asp404 *, Ile441 *, Trp481, Trp516 *, Asp518 *, Met519 *, Phe525, Arg600 *, Asp616 *
**LRP**	0.722289	0.001416	L1, R2, P3	Asp282, Trp376 *, Asp404 *, Ile441 *, Trp481, Trp516 *, Asp518 *, Met519 *, Phe525, Arg600 *, Asp616 *
**FE**	0.589707	0.1065	F1, E2	Trp376 *, Asp404 *, Ile441 *, Trp481, Trp516 *, Asp518 *, Trp613 *, Asp616 *, Phe649 *, His674 *
**RPH**	0.582675	0.0001519	R1, P2, H3	Asp282, Trp376 *, Asp404 *, Ile441 *, Trp481, Asp518 *, Met519 *, Phe525, Arg600 *, Asp616 *, Phe649 *
**YL**	0.57536	0.1541	Y1, L2	Trp376 *, Asp404 *, Ile441 *, Trp481, Asp518 *, Met519 *, Asp616 *, Phe649 *
**LR**	0.569984	0.009125	L1, R2	Trp376 *, Asp404 *, Ile441 *, Trp481, Trp516 *, Asp518 *, Asp616 *, Phe649 *, His674 *
**RR**	0.565498	0.001149	R1, R2	Trp376 *, Asp404 *, Ile441 *, Trp481, Trp516 *, Asp518 *, Met519 *, Arg600 *, Asp616 *, Phe649 *, His674 *
**NVLSGFDPQF**	0.514393	0.006611	N1, L3, D10, P8, Q9, F10	Asp282, Trp376 *, Asp404 *, Ile441 *, Trp481, Asp518 *, Met519 *, Phe525, Arg600 *, Asp616 *, Trp618

* Binding site for α-glucosidase inhibitors; peptides in bold are those selected for molecular docking.

**Table 4:** Biologically active peptides from a selected lupin protein hydrolysate (ACT) and their binding potential with α-amylase (1SMD) as a protein receptor.

Peptide Sequence	Peptide Ranker Score	PepSite2 *p*-Value	Reactive Residues in Peptide	Bound Residues of α-Amylase (1SMD)
**FP**	0.993916	0.0001681	F1, P2	Trp58 *, Trp59 *, Asp300 *
**RW**	0.978386	0.01257	R1, W2	His15, Gln41, Trp58 *, Tyr62 *, Arg195 *, Asn298, His299 *, Asp300 *, Arg337
**SPRRF**	0.919692	0.0007243	P2, R3, R4, F5	His15, Phe17, Gln41, Val42, Ser43, Pro44, Trp58 *, Trp59 *, Tyr62 *, Asp96 *, His299 *, Asp300 *
**PMLL**	0.89733	0.00183	P1, M2, L3, L4	Phe17, Glu18, Trp58 *, Trp59 *, Tyr62 *, His299 *, Asp300 *, Tyr342
**ML**	0.894564	0.02935	M1, L2	Trp58 *, Trp59 *, Tyr62 *, His299 *, Asp300 *
**AIPINNPGKL**	0.807084	0.003841	I2, P3, I4, N5, N6, P7	Phe17, Trp58 *, Trp59 *, Tyr62 *, His299 *, Asp300 *
**MLLL**	0.756994	0.03077	M1, L2, L3, L4	His15, Phe17, Glu18, Gln41, Val42, Ser43, Pro44, Trp58 *, Trp59 *, Tyr62 *, Asp96 *, His299 *, Asp300 *, Tyr342
**AIPPGIPY**	0.753808	0.01494	I2, P3, P4, P7, Y8	Phe17, Trp58 *, Trp59 *, Tyr62 *, His299 *, Asp300 *, His305 *
**HSDADFIL**	0.683272	0.0521	A4, D5, F6, I7, L8	Phe17, Trp58 *, Trp59 *, Tyr62 *, His299 *, Asp300 *
**RLL**	0.607206	0.022	R1, L2, L3	Trp58 *, Trp59 *, Tyr62 *, His299 *, Asp300 *
**FE**	0.589707	0.08928	F1, E2	His15, Gln41, Val42, Ser43, Pro44, Tyr62 *, Asp96 *, Arg195 *, His299 *, Arg337
**LR**	0.569984	0.0167	L1, R2	Trp58 *, Trp59 *, Tyr62 *, His299 *, Asp300 *
**RR**	0.565498	0.003765	R1, R2	Phe17, Trp58 *, Tyr62 *, His299 *, Asp300 *
**LRL**	0.564172	0.02945	L1, R2, L3	Phe17, Glu18, Trp58 *, Trp59 *, Tyr62 *, His299 *, Asp300 *, Tyr342
**SVPGCT**	0.501911	0.003133	V2, P3, G4, C5, T6	Phe17, Trp58 *, Trp59 *, Tyr62 *, His299 *, Asp300 *, His305 *, Lys352, Asp356
**LLPH**	0.501446	0.001419	L1, L2, P3, H4	Phe17, Glu18, Trp58 *, Trp59 *, Tyr62 *, His299 *, Asp300 *, Tyr342

* Binding site for α-amylase inhibitors; peptides in bold are those selected for molecular docking.

**Table 5:** Biologically active peptides from a selected lupin protein hydrolysate (FCT) and their binding potential with α-amylase (1SMD) as a protein receptor.

Peptide Sequence	Peptide Ranker Score	PepSite2 *p*-Value	Reactive Residues in Peptide	Bound Residues of α-Amylase (1SMD)
**FP**	0.993916	0.0001681	F1, P2	Trp58 *, Trp59 *, Asp300 *
**ML**	0.894564	0.02935	M1, L2	Trp58 *, Trp59 *, Tyr62 *, His299 *, Asp300 *
**PPGIP**	0.838217	0.001008	P1, P2, G3, I4	Trp58 *, Trp59 *, Asp300 *, HIs305 *
**TF**	0.826678	0.02906	T1, F2	Trp58 *, Trp59 *, Asp300 *
**AIPINNPGKL**	0.807084	0.003841	I2, P3, I4, N5, N6, P7	Phe17, Trp58 *, Trp59 *, His299 *, Asp300 *
**LP**	0.79612	0.0001394	L1, P2	Trp58 *, Trp59 *, Tyr62 *, His299 *, Asp300 *
**RPR**	0.722632	0.0001414	R1, P2, R3	Phe17, Trp58 *, Trp59 *, Tyr62 *, His299 *, Asp300 *
**LRP**	0.722289	0.0003887	L1, R2, P3	Phe17, Glu18, Trp58 *, Trp59 *, Tyr62 *, His299 *, Tyr342
**FE**	0.589707	0.08928	F1, E2	His15, Gln41, Val42, Ser43, Pro44, Tyr62 *, Asp96 *, Arg195 *, His299 *, Arg337
**RPH**	0.582675	0.0002215	R1, P2, H3	Trp58 *, Trp59 *, Tyr62 *, Asp300 *
**YL**	0.57536	0.03237	Y1, L2	Trp58 *, Trp59 *, His299 *, Asp300 *
**LR**	0.569984	0.0167	L1, R2	Trp58 *, Trp59 *, His299 *, Asp300 *
**EGDIIAIPPGIP**	0.568358			
**RR**	0.565498	0.003765	R1, R2	Phe17, Trp58 *, Tyr62 *, His299 *, Asp300 *
**NVLSGFDPQF**	0.514393	0.01987	V2, L3, S4, F6, P8, F10	His15, Gln41, Val42, Ser43, Pro44, Trp58 *, Trp59 *, Tyr62 *, Asp96 *

* Binding site for α-amylase inhibitors; peptides in bold are those selected for molecular docking.

**Table 6:** Binding affinity and some physicochemical properties of the selected α-glucosidase and α-amylase inhibitors from ACT and FCT hydrolysates.

Sequence	Binding Affinity (kcal/mol)	Hydrophobicity	Hydrogen Bond	Hydrophobic Interaction
**α-glucosidase**				
**RPR**	−6.1	+11.66	Asp616	Ser676, Leu650, Phe649, Leu678, Trp376 *
**LRP**	−6.2	+8.60	Asp616	Leu650, Ser676, Trp376 *, Trp481, Asp282, Phe525
**RR**	−6.4	+11.52	Asp616, Asp518	Trp376 *, Leu650, Phe649 *, Trp613
**SPRRF**	−6.6	+10.41	Arg411	Leu677, Ser379, Asp404 *, Asn417, Leu405, Asp419, Met408, Ser410
**FE**	−6.0	+9.82	ND	Arg600, Leu650, Phe649 *, Asp616 *, Trp376 *, Trp481, Met519
**PPGIP**	−5.3	+8.35	ND	Leu650, Leu678, Trp376 *, Trp481
**α-amylase**				
**ML**	−6.9	+5.98	Asp300	Asp356, Trp58 *, Trp59 *, Tyr62 *, Asp197, His305
**LP**	−7.0	+6.79	Trp59, His101	Ser163, Leu162, Leu165, Gln63, Tyr62 *, Asp197, Asp300 *
**MLLL**	−8.2	+3.48	Asp300	Lys352, Asp356, His305, Ile235, Gly306, His299 *, Glu233, Asp197, Gln63, Tyr62 *, Leu165, Trp58 *, Trp59 *
**SPRRF**	−9.1	+10.41	His305, Asp356, Tyr151, Gly306, Asp96, His299, Glu233, Asp300, Asp197	Ser163, Leu162, Leu165, Trp58 *, Trp59 *, Tyr62 *, Arg195
**AIPPGIPY**	−8.4	+7.02	Trp59, Ala106	Asp356, Trp357, Asp353, His305 *, Trp58 *, Ser163, Leu165, Gln63, Gly104, Pro54
**RPR**	−8.2	+11.66	Asp300, His305	Trp59 *, His299 *, Tyr62 *, Glu233, Leu162, Tyr151, Leu165, His201, Ile235, Asp197, Ala198

* Potential hotspots that could inhibit α-glucosidase and α-amylase activity if bounded by the peptide.

## Data Availability

Not applicable.

## Supplementary Materials

The following supporting information can be downloaded at: https://www.mdpi.com/article/10.3390/foods11213375/s1, Table S1: Degree of hydrolysis and protein content of lupin protein isolate (LPI) and its hydrolysate, Table S2: List of α-amylase and α-glucosidase inhibitory peptides identified in lupin protein hydrolysate and prepared using alcalase (ACT), Table S3: List of α-amylase and α-glucosidase inhibitory peptides identified in lupin protein hydrolysate and prepared using flavourzyme (FCT).

## References

[1] IDF (2021). Diabetes Atlas Tenth Edition.

[2] Famuwagun A.A., Alashi A.M., Gbadamosi O.S., Taiwo K.A., Oyedele D., Adebooye O.C., Aluko R.E. (2021). Antioxidant and enzymes inhibitory properties of Amaranth leaf protein hydrolyzates and ultrafiltration peptide fractions. J. Food Biochem..

[3] Patil, Mandal S., Tomar S.K., Anand S. (2015). Food protein-derived bioactive peptides in management of type 2 diabetes. Eur. J. Nutr..

[4] Fadimu G.J., Gill H., Farahnaky A., Truong T. (2022). Improving the enzymolysis efficiency of lupin protein by ultrasound pretreatment: Effect on antihypertensive, antidiabetic and antioxidant activities of the hydrolysates. Food Chem..

[5] O’Brien M.J., Karam S.L., Wallia A., Kang R.H., Cooper A.J., Lancki N., Moran M.R., Liss D.T., Prospect T.A., Ackermann R.T. (2018). Association of second-line Antidiabetic medications with cardiovascular events among insured adults with Type 2 diabetes. JAMA Netw. Open.

[6] Nourmohammadi E., Mahoonak A.S. (2019). Health implications of bioactive peptides: A review. Int. J. Vitam. Nutr. Res..

[7] Singh B.P., Aluko R.E., Hati S., Solanki D. (2022). Bioactive peptides in the management of lifestyle-related diseases: Current trends and future perspectives. Crit. Rev. Food Sci. Nutr..

[8] Guo X., Shang W., Strappe P., Zhou Z., Blanchard C. (2018). Peptides derived from lupin proteins confer potent protection against oxidative stress. J. Sci. Food Agric..

[9] Kamran F., Phillips M., Reddy N. (2021). Functional properties of Australian blue lupin (*Lupinus angustifolius*) protein and biological activities of protein hydrolysates. Legume Sci..

[10] Fadimu G.J., Farahnaky A., Gill H., Truong T. (2022). Influence of ultrasonic pretreatment on structural properties and biological activities of lupin protein hydrolysate. Int. J. Food Sci. Technol..

[11] Lammi C., Bollati C., Lecca D., Abbracchio M.P., Arnoldi A. (2019). Lupin peptide T9 (GQEQSHQDEGVIVR) modulates the mutant PCSK9D374Y Pathway: In vitro characterization of its dual hypocholesterolemic behavior. Nutrients.

[12] Muñoz E.B., Luna-Vital D.A., Fornasini M., Baldeón M.E., de Mejia E.G. (2018). Gamma-conglutin peptides from Andean lupin legume (*Lupinus mutabilis* Sweet) enhanced glucose uptake and reduced gluconeogenesis in vitro. J. Funct. Foods.

[13] Patil S.P., Goswami A., Kalia K., Kate A.S. (2020). Plant-derived bioactive peptides: A treatment to cure diabetes. Int. J. Pept. Res. Ther..

[14] Fadimu G.J., Gill H., Farahnaky A., Truong T. (2021). Investigating the impact of ultrasound pretreatment on the physicochemical, structural and antioxidant properties of lupin protein hydrolysates. Food Bioproc. Technol..

[15] Laemmli U. (1970). SDS-PAGE Laemmli method. Nature.

[16] Wickramaratne M.N., Punchihewa J., Wickramaratne D. (2016). In-vitro alpha-amylase inhibitory activity of the leaf extracts of Adenanthera pavonina. BMC Complement Altern. Med..

[17] Lankatillake C., Luo S., Flavel M., Lenon G.B., Gill H., Huynh T., Dias D.A. (2021). Screening natural product extracts for potential enzyme inhibitors: Protocols, and the standardisation of the usage of blanks in α-amylase, α-glucosidase and lipase assays. Plant Methods.

[18] Sarah S., Faradalila W., Salwani M., Amin I., Karsani S., Sazili A. (2016). LC–QTOF-MS identification of porcine-specific peptide in heat treated pork identifies candidate markers for meat species determination. Food Chem..

[19] Lamiable A., Thévenet P., Rey J., Vavrusa M., Derreumaux P., Tufféry P. (2016). PEP-FOLD3: Faster de novo structure prediction for linear peptides in solution and in the complex. Nucleic Acids Res..

[20] Madhavi Sastry G., Adzhigirey M., Day T., Annabhimoju R., Sherman W. (2013). Protein and ligand preparation: Parameters, protocols, and influence on virtual screening enrichments. J. Comput.-Aided Mol. Des..

[21] Honorato R.V., Koukos P.I., Jiménez-García B., Tsaregorodtsev A., Verlato M., Giachetti A., Rosato A., Bonvin A.M. (2021). Structural biology in the clouds: The WeNMR-EOSC ecosystem. Front. Mol. Biosci..

[22] Awosika T.O., Aluko R.E. (2019). Inhibition of the in-vitro activities of α-amylase, α-glucosidase and pancreatic lipase by yellow field pea (*Pisum sativum* L.) protein hydrolysates. Int. J. Food Sci. Technol..

[23] Aluko R.E. (2018). Food protein-derived peptides: Production, isolation, and purification. Proteins in Food Processing.

[24] Girgih A.T., Udenigwe C.C., Li H., Adebiyi A.P., Aluko R.E. (2011). Kinetics of enzyme inhibition and antihypertensive effects of hemp seed (*Cannabis sativa* L.) protein hydrolysates. J. Am. Chem. Soc..

[25] Wei D., Fan W., Xu Y. (2019). In vitro production and identification of angiotensin-converting enzyme (ACE) inhibitory peptides derived from distilled spent grain prolamin isolate. Foods.

[26] Trabuco L.G., Lise S., Petsalaki E., Russell R.B. (2012). PepSite: Prediction of peptide-binding sites from protein surfaces. Nucleic Acids Res..

[27] Hermans M., Kroos M., Van Beeumen J., Oostra B., Reuser A. (1991). Human lysosomal alpha-glucosidase. Characterization of the catalytic site. J. Biol. Chem..

[28] Bruckmann C., Repo H., Kuokkanen E., Xhaard H., Heikinheimo P. (2012). Systematic Structure-Activity Study on Potential Chaperone Lead Compounds for Acid α-Glucosidase. ChemMedChem.

[29] Kamal H., Mudgil P., Bhaskar B., Fisayo A.F., Gan C.-Y., Maqsood S. (2021). Amaranth proteins as potential source of bioactive peptides with enhanced inhibition of enzymatic markers linked with hypertension and diabetes. J. Cereal Sci..

[30] Roig-Zamboni V., Cobucci-Ponzano B., Iacono R., Ferrara M.C., Germany S., Bourne Y., Parenti G., Moracci M., Sulzenbacher G. (2017). Structure of human lysosomal acid α-glucosidase–a guide for the treatment of Pompe disease. Nat. Commun..

[31] Yang H., Liu L., Shin H.-d., Chen R.R., Li J., Du G., Chen J. (2013). Structure-based engineering of histidine residues in the catalytic domain of α-amylase from Bacillus subtilis for improved protein stability and catalytic efficiency under acidic conditions. J. Biotechnol..

[32] Yadav J.K., Prakash V. (2011). Stabilization of α-amylase, the key enzyme in carbohydrates properties alterations, at low pH. Int. J. Food Prop..

[33] Buisson G., Duee E., Haser R., Payan F. (1987). Three-dimensional structure of porcine pancreatic alpha-amylase at 2.9 A resolution. Role of calcium in structure and activity. EMBO J..

[34] Siow H.-L., Gan C.-Y. (2016). Extraction, identification, and structure-activity relationship of antioxidative and α-amylase inhibitory peptides from cumin seeds (*Cuminum cyminum*). J. Funct. Foods.

[35] Yu X., Cai X., Li S., Luo L., Wang J., Wang M., Zeng L. (2022). Studies on the interactions of theaflavin-3, 3′-digallate with bovine serum albumin: Multi-spectroscopic analysis and molecular docking. Food Chem..

[36] Park H., Hwang K.Y., Oh K.H., Kim Y.H., Lee J.Y., Kim K. (2008). Discovery of novel α-glucosidase inhibitors based on the virtual screening with the homology-modeled protein structure. Bioorg. Med. Chem..

[37] Siow H.-L., Lim T.S., Gan C.-Y. (2017). Development of a workflow for screening and identification of α-amylase inhibitory peptides from food source using an integrated Bioinformatics-phage display approach: Case study–Cumin seed. Food Chem..

