# Supplementary material for: In-Silico Analysis and Antidiabetic Effect of α-Amylase and α-Glucosidase Inhibitory Peptides from Lupin Protein Hydrolysate: Enzyme-Peptide Interaction Study Using Molecular Docking Approach
Source: Foods. 2022 Oct 26;11(21):3375. doi: 10.3390/foods11213375 (PMC9656729; doi:10.3390/foods11213375)
Supplement: Supplementary file 1 [file foods-11-03375-s001.zip › foods-1906146-supplementary.pdf]

Supplementary Table S1: Degree of hydrolysis and protein content of lupin protein isolate (LPI) and its hydrolysate

	**Degree of hydrolysis (%)**	**Protein content (%)**
**LPI**	-	92.10±0.70
**ACT**	41.28±0.94	83.07±0.13
**FCT**	45.47±0.63	81.24±1.58

ACT: Alcalase hydrolysate; FCT: Flavourzyme hydrolysate

Supplementary Table S2

List of α-amylase and α-glucosidase inhibitory peptides identified in lupin protein hydrolysate and prepared using alcalase (ACT)

**Peptide**	**Peptide Ranker**
FP	0.993916
RW	0.978386
SPRRF	0.919692
PMLL	0.89733
ML	0.894564
AIPINNPGKL	0.807084
MLLL	0.756994
AIPPGIPY	0.753808
HSDADFIL	0.683272
RLL	0.607206
FE	0.589707
LR	0.569984
RR	0.565498
LRL	0.564172
SVPGCT	0.501911
LLPH	0.501446
QGAGG	0.408522
TLPILR	0.398038
ILNPDDNQNLR	0.392508
REGDIIAIPPGIPY	0.389645
CAGV	0.373811
QGNSVFN	0.373698
AFNVDEEIINRL	0.345798
IAFKT	0.313618
YIAGN	0.269281
LLGV	0.246104
LTLPI	0.245968
IISPK	0.233237
LDTTSLLNQLDPSPRRFYIA	0.198992

RGQPE	0.196585
YD	0.186864
NSNRPE	0.17961
VR	0.14691
NALEPDNRVE	0.125121
VR	0.114691
LAATS	0.0814739
EDNVI	0.0656674
EETICTA	0.0628415
VK	0.03329

Supplementary Table S3

List of α-amylase and α-glucosidase inhibitory peptides identified in lupin protein hydrolysate and prepared using alcalase (FCT)

**Peptide**	**Peptide Ranker**
FP	0.99392
ML	0.89456
PPGIP	0.83822
TF	0.82668
AIPINNPGKL	0.80708
LP	0.79612
RPR	0.72263
LRP	0.72229
VGNAALPPGLPR	0.69474
FE	0.58971
RPH	0.58268
YL	0.57536
LR	0.56998
EGDIIAIPPGIP	0.56836
RR	0.5655
CVVLLALPPVAPR	0.53394
EGDIIAIPPGIPY	0.52435
NVLSGFDPQF	0.51439
GCFAST	0.49805
DP	0.49056
SGNQGP	0.46846
QFLTQAFNVDEEIINRLQNPDERLK	0.43555
REGDIIAIPPGIP	0.4322
AR	0.39458
REGDIIAIPPGIPY	0.38965
IIRVEEGLGVISPKW	0.3876
CAGV	0.37381
VAHAFN	0.33398
HR	0.33151

NILSGFDPQFLSQALNIDE	0.30077
FPGSIEDVER	0.29937
EPKGL	0.29619
QR	0.27442
QVFRGIPAEVLA	0.22197
HH	0.21622
AEHGSIYKN	0.21496
KR	0.21307
NVLSGFDPQFLTQAFNVDEEIINRLQNPDERLK	0.19595
EPDNR	0.19543
QQQGA	0.19281
IIRVEEGLGVISPK	0.17558
LK	0.17004
DEFLEEAFSVDR	0.16995
KHR	0.16821
AGVALS	0.15853
QFLSQALNIDE	0.1539
DH	0.14798
RRKK	0.14223
QVVDC	0.13374
REGDIIAVPT	0.12965
EIINRLQNPDERLK	0.12656
GQEQSHQDEGVIV	0.12566
VDEEIINR	0.12189
LAAEHGSI	0.11983
EQSHQDEGVIV	0.11202
KH	0.11036
AK	0.09791
NDQATT	0.09133
ISTAN	0.09133
TTSP	0.08736
YEEL	0.0765
SK	0.07327
EGLKVIS	0.07159
TLIDTT	0.06028

TSTRV	0.05843
VEGEGA	0.05727
DT	0.05156
VK	0.03329
